# Novel CAR‐T Cells Specifically Targeting SIA‐CIgG Demonstrate Effective Antitumor Efficacy in Bladder Cancer

**DOI:** 10.1002/advs.202400156

**Published:** 2024-08-23

**Authors:** Mengting Ding, Jiaxing Lin, Caipeng Qin, Yuhao Fu, Yiqing Du, Xiaoyan Qiu, Ping Wei, Tao Xu

**Affiliations:** ^1^ Department of Urology Peking University People's Hospital Beijing 100044 China; ^2^ Center for Cell and Gene Circuit Design CAS Key Laboratory of Quantitative Engineering Biology Shenzhen Institute of Synthetic Biology Shenzhen Institute of Advanced Technology Chinese Academy of Sciences Shenzhen Guangdong 518055 China; ^3^ Department of Immunology School of Basic Medical Sciences Peking University Beijing 100191 China

**Keywords:** bladder cancer, CAR‐T cell therapy, combination therapy, SIA‐CIgG, vorinostat

## Abstract

Chimeric Antigen Receptor (CAR) T‐cell therapy is a promising cancer treatment method. However, its application in bladder cancer (BC) remains limited, partially because of the absence of appropriate target molecules. Sialylated cancer‐derived IgG (SIA‐CIgG) is highly expressed in BC and is closely associated with malignant biological behavior. However, its potential as a target for CAR‐T cell therapy to treat BC is yet to be established. Here, it is found that SIA‐CIgG is highly expressed in most BC samples but displayed limited expression in normal tissues. CAR‐T cells specifically targeting SIA‐CIgG can effectively lyse BC cells and the cytotoxicity depends on SIA‐CIgG expression. Furthermore, SIA‐CIgG CAR‐T cells demonstrate milder tumor cell lysis and enhanced persistence compared with human epidermal growth factor receptor 2 (HER2) CAR‐T cells, which have undergone extensive clinical trials. After repeated tumor antigen challenges, SIA‐CIgG CAR‐T cells display substantial alterations in both the transcriptome and chromatin accessibility. When combining SIA‐CIgG CAR‐T cell therapy with FDA‐approved drugs to treat BC, the histone deacetylase inhibitor (HDACi), vorinostat, is found to enhance the ablility of CAR‐T cells for tumor cell lysis. Therefore, the combination of SIA‐CIgG CAR‐T cells and vorinostat is promising for BC treatment.

## Graphical abstract

In this study, CAR‐T cells that specifically targeting sialylated cancer‐derived IgG (SIA‐CIgG) are constructed and optimized. The cell lysis efficiency, cytokine secretion level, immunophenotype, transcriptome, and chromatin profile of CAR‐T cells after tumor clearance are assessed. SIA‐CIgG CAR T cells are effective in treating bladder cancer (BC) in vivo, and vorinostat can improve the antitumor effects.

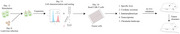

## 1 Introduction

Bladder cancer (BC) is the tenth most commonly diagnosed malignant tumor worldwide, with ≈573 000 new cases and 212 000 deaths annually.^[^ ^1^ ^]^ The subtypes of BC include urothelial carcinoma, squamous cell carcinoma, and adenocarcinoma, with urothelial carcinoma accounting for over 90% of BC cases.^[^ ^2^, ^3^ ^]^ The standard treatment regimens include surgery, radiotherapy, chemotherapy, and intravesical Bacillus Calmette–Guérin instillation.^[^ ^2^, ^4^, ^5^ ^]^ In recent years, the emergence of immune checkpoint inhibitors (ICI) and antibody‐drug conjugates that specifically target nectin‐4 have changed the BC treatment regimen.^[^ ^6^, ^7^, ^8^ ^]^ Although we have gained some understanding of the molecular mechanisms that drive the occurrence, metastasis, and drug resistance of BC,^[^ ^9^, ^10^ ^]^ the recurrence rate remains high in patients with non‐muscle invasive BC, and patients with muscle‐invasive BC have high mortality rates.^[^ ^11^ ^]^ Therefore, it is of utmost importance to explore further innovative strategies to achieve optimal treatment outcomes and improve patients' prognoses.

Chimeric antigen receptor T‐cell (CAR‐T) immunotherapy, stands out among numerous therapies due to its remarkable efficacy in B‐cell malignancies.^[^ ^12^, ^13^ ^]^ CAR‐T cell involves the genetic modification of primary human T cells to express fusion proteins composed of an extracellular ligand‐binding domain, a transmembrane region, one or two intracellular costimulatory domains, and a CD3ζ signaling domain.^[^ ^12^, ^14^ ^]^ Upon administration, these modified T cells identify specific tumor antigens and initiate a tumor‐specific immune response, leading to the eradication of tumor cells.^[^ ^12^, ^15^ ^]^ Two preclinical studies have shown the potential applications of CAR‐T cells in BC. Grunewald et al. demonstrated the efficacy of epithelial growth factor receptor (EGFR) and CD44V6‐targeting CAR‐T cells in lysing BC cells, and highlighted the potential of decitabine, a DNA methyltransferase inhibitor, in enhancing the antitumor effects.^[^ ^16^ ^]^ The other study revealed the cytotoxicity of MUC1‐targeting CAR‐T cells on BC organoids.^[^ ^17^ ^]^ Moreover, several ongoing clinical trials of CAR‐T cell therapy against BC are being conducted targeting prostate specific membrane antigen (PSMA) and folate receptor alpha (FRα), HER2 or tyrosine kinase‐like orphan receptor 2 (ROR2).^[^ ^14^ ^]^ However, till date, there are no clear outcomes from the studies.

Target selection is importance in CAR‐T cell immunotherapy; However, there are few specific targets in BC.^[^ ^18^, ^19^ ^]^ According to the classical theory, IgG production is exclusive to B cells (B cell‐derived IgG, B‐IgG). However, several studies have shown that some epithelial tumors and tumor cell lines, including BC,^[^ ^20^, ^21^ ^]^ kidney cancer,^[^ ^22^ ^]^ prostate cancer,^[^ ^23^, ^24^ ^]^ lung cancer,^[^ ^25^ ^]^ and breast cancer,^[^ ^26^ ^]^ can produce IgG (Cancer cell‐derived IgG, CIgG). Tumor cells secrete CIgG to promote their malignant behaviors or localize it on the cell membrane for mediating cell‐to‐cell interactions.^[^ ^27^ ^]^ Our previous studies revealed that CIgG can engage with integrins on the cell surface, triggering downstream pathways like FAK to facilitate tumor progression.^[^ ^28^ ^]^ Additionally, CIgG can interact with immune cell receptors, including Siglecs on T cells, to evade immune surveillance.^[^ ^29^ ^]^ Notably, we found that RP215, a monoclonal antibody developed by Lee et al., could differentiate between CIgG and B‐IgG.^[^ ^30^ ^]^ Our study demonstrated that the epitope, specifically recognized by RP215, showcased unique N‐glycosylation and high levels of sialic acid modification at the Asn162 site within the CH1 domain, rather than the classic Asn297 site located in the CH2 domain.^[^ ^28^, ^29^ ^]^ Thus, CIgG has been re‐named as sialylated CIgG (SIA‐CIgG). Our preliminary research findings demonstrated a noteworthy elevation in the expression of SIA‐CIgG on BC tissues and cells, which was correlated with the clinical pathological features and malignant biological behaviors of BC.^[^ ^21^ ^]^ Therefore, we consider SIA‐CIgG as a promising therapeutic target for BC.

In this study, we assessed the expression levels of SIA‐CIgG in BC and various normal tissues. We then employed a single‐chain fragment variable (scFv) derived from RP215 to develop second‐generation CAR‐T cells and examined their cytotoxic effects and killing specificity in BC. In addition, we depict the transcriptional alterations and chromatin openness of SIA‐CIgG CAR‐T cells following repeated tumor cell challenges. Furthermore, we showed that vorinostat, a histone deacetylase inhibitor (HDACi), augmented the antitumor effectiveness of CAR‐T cells.

## 2 Results

### 2.1 SIA‐CIgG Exists in Most BC Tissues, While Showing Minimal Expression in a Few Normal Tissues

**Figure 1:**
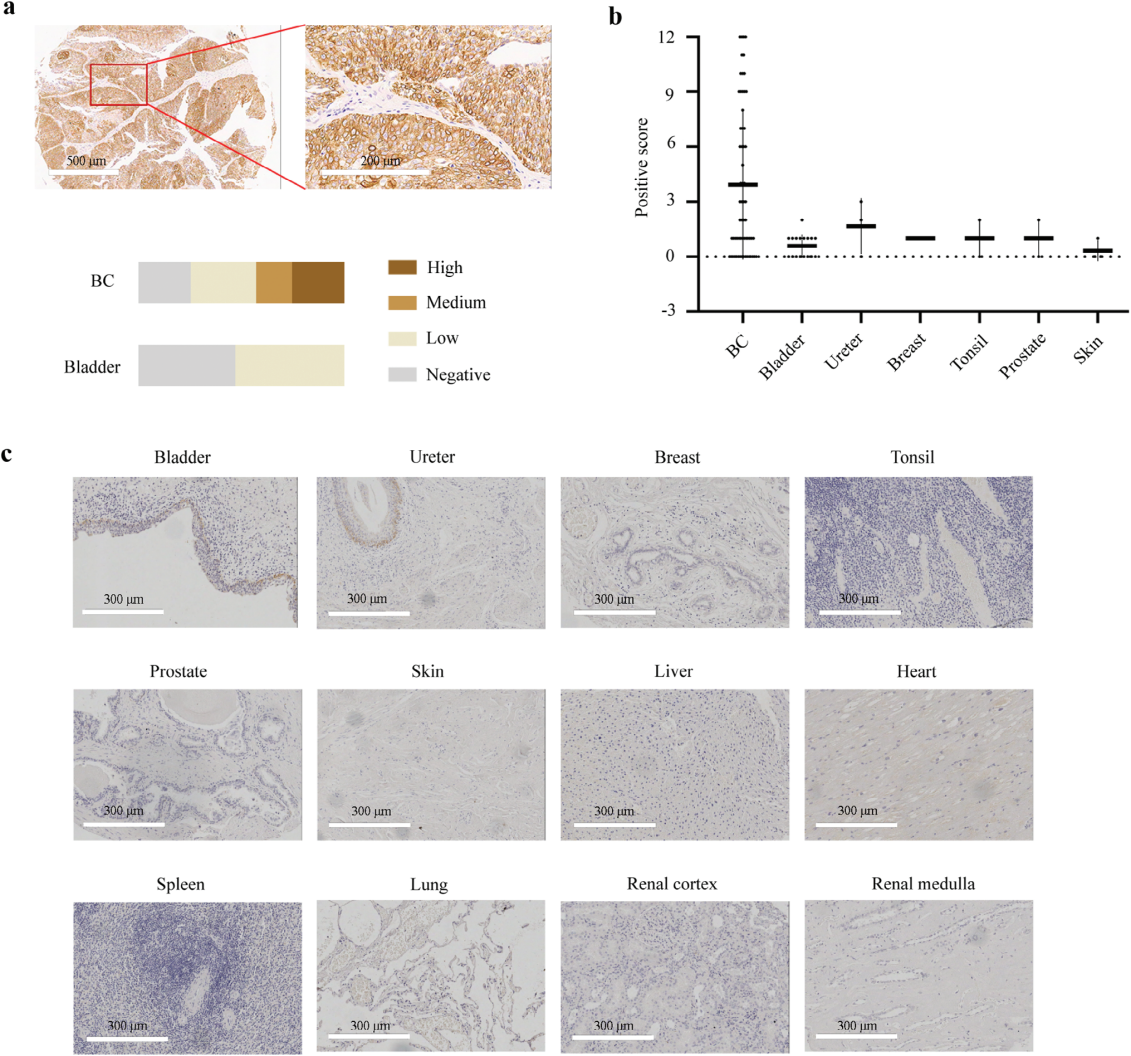
SIA‐CIgG expression in bladder cancer (BC) and normal tissues. a) Representative micrographs of SIA‐CIgG expression in BC tissues. Magnifications: 5× (left) and 20× (right). Scale bar, 500 µm (left) and 200 µm (right). The horizontal bars indicate the relative abundance of BC and normal bladder tissues based on the expression levels: negative (gray), low (light brown), medium (brown), and high (dark brown). b) Summary of the positive scores for SIA‐CIgG in BC and normal bladder tissues with SIA‐CIgG expression. c) Immunohistochemical images of normal human tissues with SIA‐CIgG, and vital human organs without SIA‐CIgG. Magnification: 10× (left). Scale bar, 300 µm.

The expression levels of SIA‐CIgG were evaluated in 63 BC and 36 types of normal tissues using immunohistochemistry. SIA‐CIgG expression was observed in approximately three‐fourths of the BC tissues, and the prevalence of low, intermediate, and high expression was 31.7%, 17.5%, and 25.4%, respectively (**Figure** **1**a,b; Table S1, Supporting Information). These findings were generally in line with our previous study, which included 77 patients diagnosed with BC.^[^ ^21^ ^]^ Therefore, we integrated data from two studies and analyzed the numbers and proportions of BC cases with low, medium, and high expression of SIA‐CIgG (Table S2, Supporting Information). We also found a significant correlation between the expression level of SIA‐CIgG and both the histological grade and the tumor, node, metastasis (TNM) stage of BC. In contrast, SIA‐CIgG expression was not correlated with age, sex, or lymph node metastasis (**Table** **1**). Moreover, we investigated the expression levels of SIA‐CIgG in 17 normal bladder tissue samples comprising 14 paired adjacent non‐cancerous samples. The results showed that 47.1% of the population had no detectable SIA‐CIgG expression, whereas the remaining individuals showed low expression (Figure 1b,c; Table S1, Supporting Information). Furthermore, we assessed SIA‐CIgG expression in additional 35 types of normal tissue types (Figure 1b,c; Figure S1, Supporting Information). Our findings revealed low expression of SIA‐CIgG in certain tissues, such as the ureter, breast, tonsils, prostate, and skin, whereas SIA‐CIgG was not detected in most normal tissues, particularly in vital organs such as the heart, lungs, spleen, kidneys, and liver.

**Table 1:** Correlation analysis of SIA‐CIgG immunostaining and clinicopathological features in patients with BC.

Characteristics	Total	SIA‐CIgG expression				P value
		Negative	Low	Medium	High	
Gender						
Female	10	4 (40.0%)	2 (20.0%)	1 (10.0%)	3 (30.0%)	0.574
Male	53	12 (22.6%)	18 (34.0%)	10 (18.9%)	13 (24.5%)	
Age						
≤60	14	2 (14.3%)	6 (42.9%)	1 (7.1%)	5 (35.7%)	0.330
>60	49	14 (28.6%)	14 (28.6%)	10 (20.4%)	11 (22.4%)	
Histological grade						
Low	8	6 (75.0%)	1 (12.5%)	1 (12.5%)	0 (0.0%)	0.006^**^
High	55	10 (18.2%)	19 (34.5%)	10 (18.2%)	16 (29.1%)	
Lymph node metastasis (N)						
N0	55	14 (25.5%)	18 (32.7%)	10 (18.2%)	13 (23.6%)	0.852
N1, N2, N3	8	2 (25.0%)	2 (25.0%)	1 (12.5%)	3 (37.5%)	
TNM stage						
0/I II/III/IV	21 42	10 (47.6%) 6 (14.3%)	7 (33.3%) 13 (31.0%)	2 (9.5%) 9 (21.4%)	2 (9.5%) 14 (33.3%)	0.015*

### 2.2 Construction and Optimization of SIA‐CIgG CAR‐T Cells

**Figure 2:**
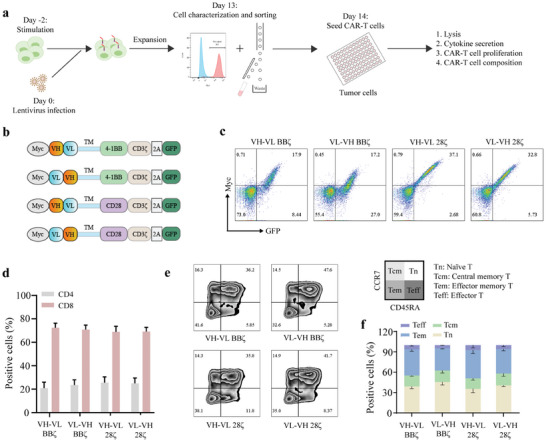
Manufacturing and characterization of SIA‐CIgG CAR‐T cells. a) Flowchart illustrating the manufacturing process and the evaluation scheme for assessing the cytotoxic effects of CAR‐T cells. b) Schematic representation of the structure of SIA‐CIgG‐targeting CARs. c) CAR transduction efficiency was assessed 48‐h post‐lentiviral infection by flow cytometry. d) The ratio of CD4+ to CD8+ T cells was determined after ≈2 weeks of in vitro culture of CAR‐T cells. e) Surface expression of CD45RA and CCR7 to differentiate naïve T cells (CD45RA^+^CCR7^+^, Tn), central memory T cells (CD45RA^−^CCR7^+^, Tcm), effector memory T cells (CD45RA^−^CCR7^−^, Tem), and effector T cells (CD45RA^+^CCR7^−^, Teff). f) The Proportion of Tn, Tcm, Tem, and Teff subsets in the total cell population.

The manufacturing process and functional evaluation of CAR‐T cells are described (**Figure** **2**a). The monoclonal antibody, RP215‐derived scFv, was used to generate CAR‐T cells against the SIA‐CIgG. Four types of CAR structures were constructed based on different configurations of the variable heavy and light chains (VH‐VL/VL‐VH) and costimulatory domains (4‐1BB or CD28) (Figure 2b). After 48 h of lentiviral infection, the transduction efficiency of T cells was assessed using flow cytometry, which typically ranged from 20–50% (Figure 2c). After a 2‐week expansion of CAR‐T cells in vitro, we evaluated the composition of the CAR‐T cell population. The results showed that CD8+ T cells comprised ≈80% of the total cell population (Figure 2d). The number of naïve T cells (Tn) and effector memory T cells (Tem) was higher than that of central memory T cells (Tcm) and effector cells (Teff) (Figure 2e,f).

**Figure 3:**
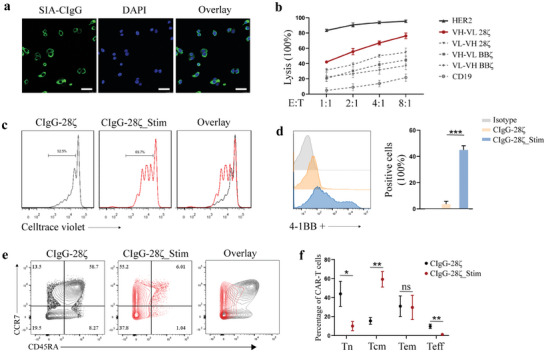
SIA‐CIgG CAR‐T cells induced tumor‐specific immune responses upon exposure to tumor antigens. a) Cell immunofluorescence staining was performed to confirm the expression of SIA‐CIgG on the cell membrane of 5637 cells. b) Specific lysis of 5637 (luciferase+) cells after co‐culture with CAR‐T cells for 24 h under different effector‐to‐target (E:T) ratios. c–f) SIA‐CIgG CAR‐T (CIgG‐28ζ) cells were initially co‐cultured with 5637 cells at a 1:1 E:T ratio, the tumor cells were replaced every 2 days, and the subsequent experiments were conducted after 6 days of continuous co‐culture. At this stage, SIA‐CIgG CAR‐T cells are regarded as stimulated cells (CIgG‐28ζ_Stim). The CAR‐T cells were initially labeled with CellTrace violet. After 6 days of co‐culture, the extent of dye dilution was assessed (c). The positivity rate of the activation marker 4‐1BB in CD8+ T cells of both unstimulated and stimulated CAR‐T cells is shown in (d). The proportions of Tn, Tcm, Tem, and Teff cells in unstimulated and stimulated CAR‐T cells were assessed (e and f). ^*^ *p* < 0.05, ^**^ *p* < 0.01, and ^***^ *p* < 0.001, “ns” means “not significant” (*p* > 0.05).

Since SIA‐CIgG exists in two forms, a secreted protein, and a membranous protein, we conducted immunofluorescence staining to identify the cell lines expressing SIA‐CIgG on the cell membrane. Our results indicated that of the seven tested BC cell lines, only 5637 exhibited strong positive expression of SIA‐CIgG on the cell membrane (**Figure** **3**a; Figure S2, Supporting Information). Therefore, it was selected for subsequent CAR‐T cell cytotoxicity experiments. We conducted a cytolytic assay to assess the killing efficiency of the four CAR‐T cell types. Here, second‐generation HER2 and CD19 CAR‐T cells with the CD28 costimulatory domain served as controls. We found that the lytic capacity of CAR‐T cells toward tumor cells increased with increasing dosage. Notably, among the four types of SIA‐CIgG‐targeting CAR‐T cells, the one possessing a VH‐VL structure and a CD28 costimulatory domain demonstrated superior killing capacity (Figure 3b). Hence, this type of CAR‐T cells (referred to as CIgG‐28ζ) was used in all subsequent experiments. Notably, HER2 CAR‐T cells displayed a robust capability for tumor cell eradication even at a relatively low effector‐to‐target ratio (E:T = 1:1) (Figure 3b).

### 2.3 SIA‐CIgG CAR‐T Cells Could Elicit Tumor‐Specific Immune Responses Upon Exposure to Tumor Antigens

Alterations in T cells following infection with the CAR lentivirus targeting SIA‐CIgG were analyzed after three rounds of tumor cell killing. We found an increase in the proportion of CAR‐T cells within the entire cell population and a concurrent increase in the mean fluorescence intensity (MFI) of GFP compared with CAR‐T cells not exposed to tumor cells (Figure S3a, Supporting Information), indicating that SIA‐CIgG CAR‐T cells exhibited a proliferative response upon contact with tumor cells. Similarly, we labeled CAR‐T cells with CellTrace Violet dye and observed the expansion of CAR‐T cells following their interaction with tumor cells (Figure 3c). Owing to the limited number of CD4+ T cells during this period (Figure S3b, Supporting Information), we detected the expression levels of the activation marker 4‐1BB in CD8+ CAR‐T cells and observed noteworthy upregulation (Figure 3d). Importantly, following tumor cell lysis, SIA‐CIgG CAR‐T cells exhibited a complete transition into the Tcm and Tem phenotypes (Figure 3e), whereas we did not observe such a significant change in non‐transduced T cells (Figure S3c, Supporting Information). In conclusion, SIA‐CIgG CAR‐T cells are functionally competent and capable of inducing tumor‐specific immune responses.

### 2.4 SIA‐CIgG CAR‐T Cells Demonstrated Milder Cytotoxic Effects and Superior Persistence Compared with HER2 CAR‐T Cells

**Figure 4:**
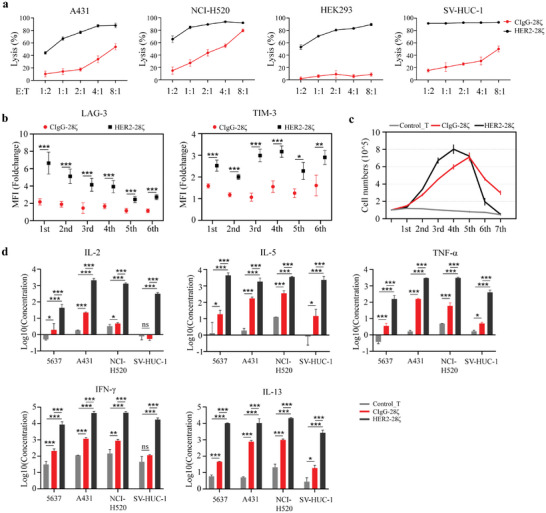
Comparison of the performance of SIA‐CIgG CAR‐T cells and HER2 CAR‐T cells following tumor antigen stimulation. a) Specific lysis of tumor and non‐tumor cells (luciferase+) after co‐culture with CAR‐T cells targeting SIA‐CIgG or HER2 for 24 h at different E:T ratios. b) Fold changes in the mean fluorescence intensity (MFI) of LAG‐3 and TIM‐3 on the surface of stimulated SIA‐CIgG and HER2 CAR‐T cells compared to unstimulated CAR‐T cells after six rounds of tumor challenge. c) Cell proliferation of SIA‐CIgG and HER2 CAR‐T cells after multiple rounds of tumor challenge. d) Secretion of IL‐2, IL‐5, TNF‐α, IFN‐γ, and IL‐13 by SIA‐CIgG CAR‐T and HER2 CAR‐T cells after co‐culture with target cells at a 2:1 (E:T) ratio for 24 h. ^*^ *p* < 0.05, ^**^ *p* < 0.01, and ^***^ *p* < 0.001, “ns” means “not significant” (*p* > 0.05).

To further determine the efficiency and specificity of SIA‐CIgG CAR‐targeted cell lysis, we assessed the lytic capacity of SIA‐CIgG CAR‐T cells against SIA‐CIgG‐positive cell lines derived from other tumors (A431 and NCI‐H520), the human uroepithelial cell line SV‐HUC‐1, and SIA‐CIgG‐negative HEK293 cells (Figure S4a,b, Supporting Information). HER2 was uniformly expressed on the membranes of all tested cell lines (Figure S4c, Supporting Information). Thus, HER2 CAR‐T cells were used as positive controls. Our results indicated the potent cytolytic capacity of HER2 CAR‐T cells against all target cells, including HEK293 cells derived from normal tissues. In comparison, SIA‐CIgG CAR‐T cells presented lower cytolytic potency against A431 and NCI‐H520 cells, while displaying no apparent cytotoxicity toward the non‐tumor cell line HEK293, even at high E:T ratios (**Figure** **4**a). We observed that SIA‐CIgG CAR‐T cells exerted cytotoxicity against SV‐HUC‐1 cells to some extent, which could be attributed to the surface expression of SIA‐CIgG in a subset of SV‐HUC‐1 cells (Figure S4a, Supporting Information). In summary, SIA‐CIgG CAR‐T cells exhibit killing specificity as they lyse target cells in an SIA‐CIgG‐dependent manner.

Numerous clinical trials are currently underway to investigate the therapeutic efficacy of CAR‐T cells targeting HER2 in solid tumors.^[^ ^14^, ^31^ ^]^ We observed a significant difference in the cytotoxicity between HER2 CAR‐T and SIA‐CIgG CAR‐T cells. Therefore, a comparative analysis was performed between the two CAR‐T cell types. We dynamically monitored the responses of SIA‐CIgG and HER2 CAR‐T cells during repetitive tumor antigen stimulation. We found that HER2 CAR‐T cells acquired enhanced activation after encountering tumor antigens, as indicated by the greater upregulation of TIM‐3 and LAG‐3 (Figure 4b), along with a superior expansion potential compared to SIA‐CIgG CAR‐T cells (Figure 4c). However, because of this vigorous and prolonged activation, HER2 CAR‐T cells experienced rapid and extensive cell death after multiple rounds of cytotoxicity experiments (Figure 4c). Conversely, SIA‐CIgG CAR‐T cells manifested a reduced exhaustion rate compared with HER2 CAR‐T cells, indicating their superior persistence.

Moreover, the levels of cytokines secreted by these two types of CAR‐T cells during tumor cell elimination at the same E:T ratio (2:1) were measured. Both SIA‐CIgG CAR‐T cells and HER2 CAR‐T cells secreted more IL‐2, IL‐5, TNF‐α, IFN‐γ, and IL‐13 compared to non‐transduced T cells when eliminating tumor cells. However, HER2 CAR‐T cells produced approximately an order of magnitude greater quantities of cytokines than SIA‐CIgG CAR‐T cells (Figure 4d).

### 2.5 Analysis of the Alterations in the Transcriptome and Chromatin Accessibility of Stimulated CAR‐T Cells

**Figure 5:**
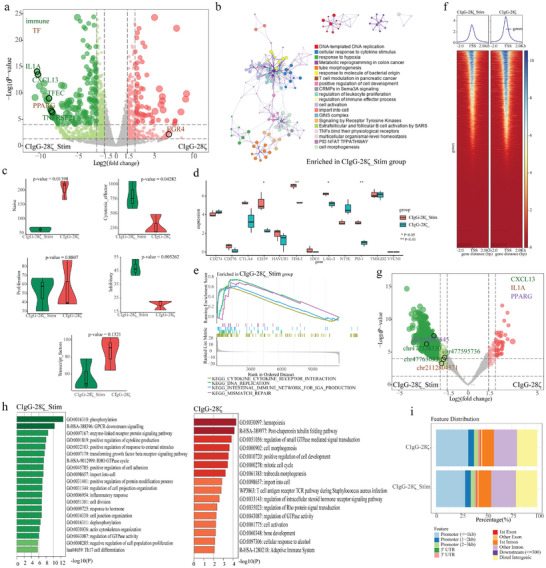
Comparison of the transcriptome and chromatin accessibility between unstimulated and stimulated CAR‐T cells. a) Differential Gene Expression Analysis in the unstimulated (CIgG‐28ζ) and stimulated groups (CIgG‐28ζ_Stim), with emphasis on the annotation of immune‐related genes and transcription factors. b) Enrichment analysis of genes highly expressed in stimulated CAR‐T cells. c) Statistical testing of T cell‐related scores between two cell groups. d) Differential expression of immune checkpoints in unstimulated and stimulated CAR‐T cells. e) Gene Set Enrichment Analysis (GSEA) of pathways in stimulated CAR‐T cells. f) Heat map of chromatin accessibility near transcription start sites (TSS) of genes. g) Comparison of gene accessibility between the two cell groups. h) Enrichment analysis of genes with high accessibility levels in the stimulated and unstimulated group. i) Distribution of chromatin‐accessible regions enriched across the genome in unstimulated and stimulated CAR‐T cells. ^*^ *p* < 0.05, and ^**^ *p* < 0.01.

To elaborate on the changes in the transcriptome and chromatin accessibility of SIA‐CIgG CAR‐T cells after eliminating tumor cells, we sorted two sets of CAR‐T cells: those that underwent continuous stimulation with tumor antigens for 14 days and a corresponding set of unstimulated CAR‐T cells from the same batch. Transcriptome analysis and ATAC sequencing were then performed. The results revealed a higher expression of immune‐related genes, such as IL1A, CXCL13, TFEC, TNFRSF21, and the transcription factor *PPARG*, in stimulated CAR‐T cells (**Figure** **5**a). Metascape enrichment analysis of stimulated CAR‐T cells identified the activation of immune‐related signaling pathways and hypoxia‐related pathways (Figure 5b). The scoring of T cell functional gene sets in both groups showed a significant increase in cytotoxicity and inhibitory scores in stimulated CAR‐T cells, whereas naïve scores significantly decreased (Figure 5c). In a study of immune checkpoints, CD39, T cell immunoglobulin domain and mucin domain‐3 (TIM‐3), lymphocyte activation gene‐3 (LAG‐3), and programmed cell death‐1 (PD‐1) were significantly upregulated in stimulated CAR‐T cells (Figure 5d). GSEA KEGG analysis of CIgG‐28ζ_Stim CAR‐T and CIgG‐28ζ CAR‐T revealed an enrichment of cytokine‐receptor interactions in stimulated CAR‐T cells (Figure 5e). The ATAC sequencing results indicated significantly lower chromatin accessibility in stimulated CAR‐T cells than in unstimulated CAR‐T cells (Figure 5f). Consistent with RNA‐seq results, differential analysis of gene accessibility signals showed higher accessibility of *CXCL13*, *IL1A*, and *PPARG* in stimulated CAR‐T cells (Figure 5g). The stimulated CAR‐T cells were likely associated with cellular responses to external signals, proliferation, and differentiation, as well as the activation of pathways related to cytokine production and immune response (Figure 5h). In the promoter (< = 1 kb) region, chromatin accessibility in stimulated CAR‐T cells was significantly lower than that in unstimulated CAR‐T cells (Figure 5i).

### 2.6 The HDACi Vorinostat Enhanced the Killing Efficacy of SIA‐CIgG CAR‐T Cells

Given its relatively weak cytotoxicity compared to that of HER2 CAR‐T cells, we speculated that the antitumor effects could be improved by combining CAR‐T cell therapy with FDA‐approved drugs. Herein, we assessed the influence of seven drugs on the antitumor effects of SIA‐CIgG CAR‐T cells, including the conventional chemotherapy agents cisplatin and gemcitabine, the tyrosine kinase inhibitor erdafitinib, the PD‐1 monoclonal antibody nivolumab, the mTOR inhibitor everolimus, the HDACi vorinostat, and a second mitochondria‐derived activator of caspases (SMAC) mimetic birinapant.

**Figure 6:**
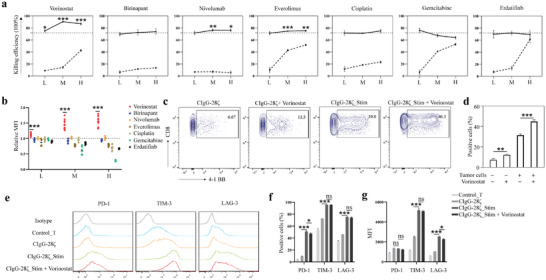
Vorinostat activated SIA‐CIgG CAR‐T cells and enhanced their cytotoxicity. a) The impact of seven drugs at three different concentrations on the cytotoxic efficacy of CAR‐T cells. The dashed lines represent the cytotoxic effects of SIA‐CIgG CAR‐T cells alone on 5637 cells, whereas the dashed broken lines indicate the effect of the drugs alone on cell viability. The solid dashed lines represent the combined effects of the drugs and CAR‐T cells on cell cytotoxicity. b) Influence of varying drug concentrations on GFP expression in SIA‐CIgG CAR‐T cells. c,d) The expression of 4‐1BB on CAR‐T cells was significantly changed after 48 h of stimulation by tumor cells in the presence of vorinostat (1 µm). e–g) The positive rates and MFI of PD‐1, TIM‐3, and LAG‐3 on CAR‐T cells were evaluated after 48 h of stimulation by tumor cells in the presence of 1 µm vorinostat. L, M, H: Low, medium, and high concentrations, respectively. ^*^ *p* < 0.05, ^**^ *p* < 0.01, and ^***^ *p* < 0.001, “ns” means “not significant” (*p* > 0.05).

We investigated the anti‐tumor effects of CAR‐T cells in combination with drugs at three different concentrations. The selection of these concentrations followed a principled approach: the low concentration exhibited negligible toxicity toward tumor cells, the high concentration displayed evident toxicity, and the moderate concentration served as an intermediate point between the two groups. Enhanced antitumor effects were observed when CAR‐T cells were combined with vorinostat, nivolumab, and everolimus (**Figure** **6**a). Next, we explored whether this phenomenon resulted from the additive effects of the drugs and CAR‐T cells, or if the drugs augmented the functionality of CAR‐T cells. We found that, after a 1‐day vorinostat treatment, there was an increase in the MFI of GFP carried by CAR‐T cells, indicating an upregulation of CAR expression on the T cell surface (Figure 6b). However, this phenomenon was not observed for other drugs tested. Furthermore, we observed significant upregulation of 4‐1BB in CD8+ CAR‐T cells following treatment with vorinostat (Figure 6c,d). Our results show that vorinostat promptly and notably enhanced the cytolytic capacity of CAR‐T cells. Moreover, there was a slight reduction in the surface expression of PD‐1 and LAG‐3 on CAR‐T cells after vorinostat treatment (Figure 6e–g). Overall, we consider vorinostat to have the potential to sensitize SIA‐CIgG CAR‐T cells.

### 2.7 The Co‐Administration of SIA‐CIgG CAR‐T Cells and Vorinostat Demonstrated Significant Efficacy in Treating BC

**Figure 7:**
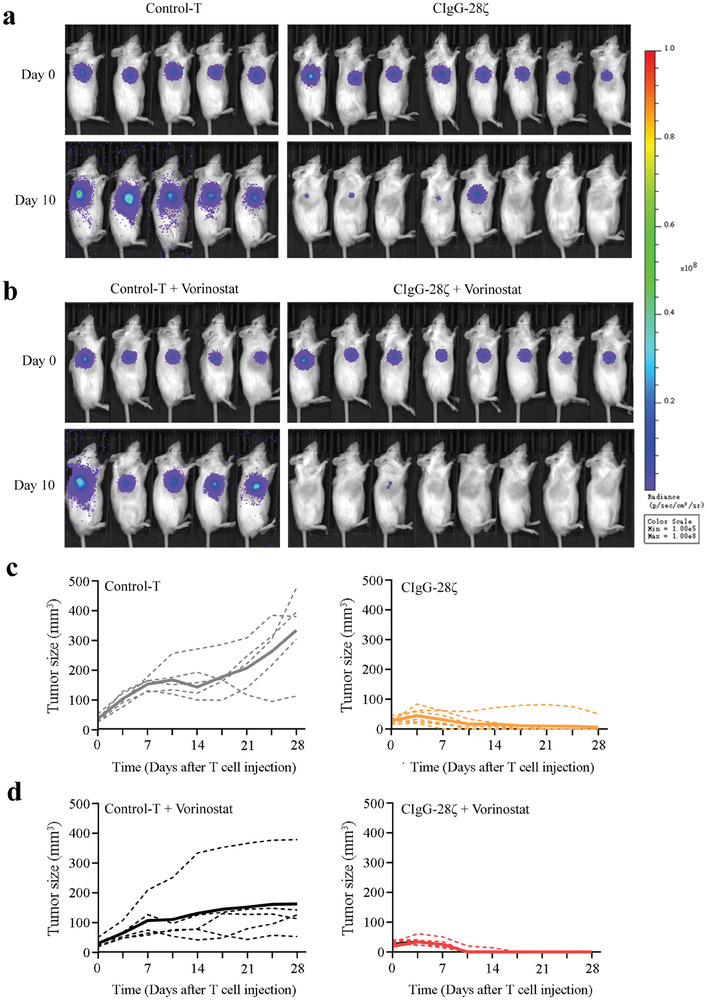
Co‐administration of SIA‐CIgG CAR‐T cells and vorinostat demonstrated effective cytotoxicity in combating BC in vivo. a) Bioluminescent images of mice before and 10 days after non‐transducted T cell (Control‐T) or SIA‐CIgG CAR‐T cell (CIgG‐28ζ) injection. b) Bioluminescent images of mice before and 10 days after non‐transducted T cell or SIA‐CIgG CAR‐T cell injection combined with vorinostat. c) Trend in tumor volume changes after injection of non‐transducted T or CAR‐T cells. d) Trend in tumor volume changes after co‐administration of non‐transducted T or CAR‐T cells and vorinostat. The tumor volumes were measured twice per week.

To determine the antitumor activity of SIA‐CIgG CAR‐T cells alone and in combination with vorinostat in vivo, BC animal models based on NOD‐SCID‐Il2rg−/− (NSG) mice were established. One week after subcutaneous inoculation of tumor cells, we divided the mice into groups based on tumor volume and in vivo imaging results. The tumors of mice receiving non‐transduced T cells (Control‐T) mostly displayed stable and continuous growth, while in the SIA‐CIgG CAR‐T cell treatment group, one, two, three, and one mouse achieved complete tumor clearance on days 7, 11, 18, and 23, respectively, following CAR‐T cell administration (**Figure** **7**a,c). In individuals receiving the combined treatment with SIA‐CIgG CAR‐T cells and vorinostat, significant tumor suppression was observed. Specifically, seven mice achieved complete tumor clearance on the 11th day following CAR‐T cell therapy, and another mouse achieved complete tumor clearance on the 18th day (Figure 7b,d). There were no signs of tumor recurrence observed in any of the mice after complete tumor clearance.

## 3 Discussion

Tumor immunotherapy is a notable breakthrough in the field of oncology. CAR‐T cell therapy has shown high efficacy in B‐cell malignancies and a possibility for treating solid tumors. Numerous clinical trials have been conducted to investigate the therapeutic effects of CAR‐T cells on solid tumors.^[^ ^31^, ^32^ ^]^ However, few have been approved for clinical applications. Several factors impede the clinical translation of CAR‐T cell therapy to solid tumors, including the absence of tumor‐specific antigens. Unlike B cell‐associated antigens such as CD19 or BCMA, where the destruction of B cells can be tolerated by patients, the presence of tumor antigens in other normal tissues can potentially cause significant side effects. BC lacks specific targets for tumor treatment. In this study, we proposed SIA‐CIgG as a promising target for CAR‐T cell therapy in treating BC.

Numerous studies have shown that SIA‐CIgG demonstrates tumorigenic effects in various types of cancers.^[^ ^27^, ^33^ ^]^ Our previous study revealed the ability of SIA‐CIgG to accelerate the progression of BC, highlighting its potential as a promising therapeutic target.^[^ ^21^ ^]^ Our findings indicated that SIA‐CIgG was present in most BC cases and that 42.9% of patients displayed moderate‐to‐high levels of SIA‐CIgG expression, whereas normal bladder tissues showed either negative or weakly positive results. Importantly, most normal tissues do not express SIA‐CIgG, particularly vital organs such as the heart, lungs, spleen, kidneys, and liver. Therefore, SIA‐CIgG CAR‐T cells exhibit commendable biological safety.

Considering the above findings, we generated four distinct types of CAR‐T cells specifically targeting SIA‐CIgG. It is well known that approved CAR‐T cell products, which target the same tumor antigen, exhibit variations in the origin of the hinge region (CD28, CD8α, or IgG4), transmembrane domain (CD28 or CD8α), or costimulatory domain (CD28 or 4‐1BB).^[^ ^13^, ^34^ ^]^ Amongst, the influence of costimulatory domains on the function of CAR‐T cells is particularly intriguing. Studies suggested that CAR‐T cells with CD28 costimulatory domains exhibit rapid expansion and reduced persistence compared with those with 4‐1BB,^[^ ^35^, ^36^ ^]^ and CD28ζ‐CARs outperform 4‐1BBζ‐CARs when antigen density is low.^[^ ^37^ ^]^ Besides, CD28‐co‐stimulated CAR‐T cells exhibit a more effector‐like memory phenotype characterized by increased glycolytic metabolism and cytokine production. In contrast, 4‐1BB‐based CAR‐T cells tend to have a central memory phenotype that depends on fatty acid metabolism.^[^ ^38^, ^39^ ^]^ Indeed, some studies suggest that CAR‐T cells containing CD28 costimulatory domains exhibit superior antitumor performance, whereas other findings indicate no significant difference or even better results with 4‐1BB‐based CAR‐T cells.^[^ ^40^, ^41^ ^]^ The underlying mechanisms have not been fully elucidated. Here, CAR‐T cells with a CD28 costimulatory domain exhibited superior therapeutic efficacy. We observed tumor‐specific immune responses in CIgG‐28ζ‐CAR‐T cells, including cell proliferation and upregulation of surface activation markers upon exposure to tumor cells. Notably, SIA‐CIgG CAR‐T cells underwent a gradual transition toward Tcm and Tem phenotypes during tumor cell lysis.

We found that SIA‐CIgG CAR‐T cells could effectively lyse SIA‐CIgG‐positive cells while causing minimal damage to SIA‐CIgG‐negative cells, indicating their specific cytotoxicity. However, the cytotoxicity and activation levels of SIA‐CIgG CAR‐T cells upon stimulation were lower than those of the HER2 CAR‐T cells. The possible reasons for this disparity include differences in tumor antigen density on the cell membrane or the affinity between scFv and tumor antigens. Nonetheless, the attenuated cytotoxicity of SIA‐CIgG CAR‐T cells contributed to their prolonged persistence and decreased cytokine secretion. The enhanced tumor specificity and persistence of SIA‐CIgG CAR‐T cells enhance their prospects for application.

In the analysis of RNA‐seq and ATAC‐seq data for CAR‐T cells subjected to long‐term and repetitive stimulation with tumor antigens, we observed the activation of immune‐related signaling pathways and hypoxia‐related pathways, as well as the enrichment of cytokine‐receptor interactions. The stimulated CAR T cells exhibited decreased overall chromatin accessibility. Notably, we observed an upregulation in the expression levels and chromatin accessibility of CXCL13 and PPARΓ in stimulated CAR‐T cells. CXCL13‐expressing T cells are tumor‐reactive T cells that are upregulated in response to T cell exhaustion.^[^ ^42^ ^]^ PPARγ plays a crucial role in directly regulating lipid metabolism in immune cells for immune activation.^[^ ^43^ ^]^

Given the relatively low lytic capacity of SIA‐CIgG CAR‐T cells, we explored their potential to augment their efficacy in combination with other therapeutic approaches. Therefore, we included drugs currently approved for BC treatment, such as cisplatin, gemcitabine, erdafitinib, and nivolumab. One previous study has demonstrated that vorinostat can effectively enhance the performance of B7‐H3 CAR‐T cells against solid tumors. Vorinostat downregulates the expression of CTLA‐4 and TET2, which are implicated in the immunosuppressive effects of CAR‐T cells.^[^ ^44^ ^]^ Likewise, birinapant is a synthetic small molecule that acts as a peptidomimetic of the second mitochondrial‐derived activator of caspases (SMAC).^[^ ^45^ ^]^ Dufva and colleagues have demonstrated that birinapant can augment the antitumor effectiveness of CD19 CAR‐T cells.^[^ ^46^ ^]^ Furthermore, numerous studies have proved the inhibitory effects of everolimus on BC.^[^ ^47^ ^]^ Therefore, we detected the killing efficiency when these drugs were combined with CAR‐T cells. Our findings demonstrate that vorinostat promotes CAR expression within T cells and enhances the activation levels of CAR‐T cells in response to stimulation by tumor cells. Co‐administration of vorinostat and CAR‐T cells yielded enhanced antitumor effects in vivo.

In conclusion, our findings confirm that SIA‐CIgG is a promising target for CAR‐T cell therapy. We constructed CAR‐T cells targeting SIA‐CIgG and demonstrated their effectiveness and specificity for BC treatment. Furthermore, we showed that SIA‐CIgG CAR‐T cells possess superior tumor specificity and persistence compared to the widely studied HER2 CAR‐T cells. In addition, we illustrated alterations in the transcriptome and chromatin accessibility of CAR‐T cells after multiple rounds of tumor cell eradication. Vorinostat enhanced the killing efficiency of SIA‐CIgG CAR‐T cells. Therefore, we believe that the combination of SIA‐CIgG CAR‐T cells and vorinostat represents a promising treatment for BC.

## 4 Experimental Section

### Cell Lines

The cell lines used in this study included the BC cell lines 5637, J82, T24, TCCSUP, SW780, EJ, and BIU‐87; the lung squamous cancer cell line NCI‐H520; the human epidermal carcinoma cell line A431; the human embryonic kidney cell line HEK293; and the human uroepithelial cell line SV‐HUC‐1. All cell lines were purchased from the American Type Culture Collection (ATCC, Manassas, VA, USA). The 5637, EJ, BIU‐87, and NCI‐H520 cell lines were cultured in RPMI 1640 medium (Gibco, Waltham, MA, USA) supplemented with 10% fetal bovine serum (FBS) (Gemini Bio, West sacramento, CA, USA) and 1% penicillin‐streptomycin (Gibco). J82, T24, TCCSUP, SW780, A431, and HEK293 cells were maintained in Dulbecco's modified Eagle medium (DMEM) (Gibco) with the same supplements. SV‐HUC‐1 cells were cultured in Ham's F‐12k medium (Gibco) supplemented with the same substances. All cell cultures were maintained in a humidity‐controlled incubator at 37 °C with 5% CO2. The 5637, NCI‐H520, A431, SV‐HUC‐1, and HEK293 cells were modified to express a luciferase (firefly) and mCherry protein vector using a lentivirus for cytotoxicity assays in vitro and in vivo.

### Immunohistochemistry

The BC tissue microarray comprised 63 BC tissues and 14 paired adjacent noncancerous tissues. It was purchased from Shanghai Xinchao Biotechnology Co., Ltd. (Shanghai, China). The normal tissue microarray consisted of 36 types of normal tissues (three samples from different individuals for each tissue type) purchased from Shanghai Wei Ao Biotechnology Co., Ltd. (Shanghai, China). The tissue microarrays underwent dewaxing and were subsequently placed in EDTA antigen retrieval solution and heated. After blocking with 10% sheep serum for 1 h, the microarrays were sequentially incubated with RP215 (4 mg mL^−1^, diluted 1:1000) and horseradish peroxidase‐conjugated goat anti‐mouse secondary antibody. The microarrays were exposed to DAB staining solution (ZSGB‐BIO, Beijing, China) for 80 s. The slides were washed thrice with PBS when necessary. IHC staining results were assessed by two experienced pathologists. The percentage of stained area was scored as follows: 0 (< 1%), 1 (1–25%), 2 (26–50%), 3 (51–75%), and 4 (75–100%). Staining intensity was scored as 0 (no staining), 1 (light brown), 2 (brown), and 3 (dark brown). Percentage and intensity scores were multiplied for each case. For RP215 staining, negative (‐) was assigned when the score was 0, “low expression” when scores were 1–3, “medium expression” when scores were 4–7, and “high expression” when scores were 8–12.

### Detection of Cell Surface Expression of SIA‐CIgG

Surface expression of SIA‐CIgG was assessed in most cells using immunofluorescence assays. The cells were seeded in a 96‐well plate for at least 1 day in advance. The supernatant was discarded, and the cells were washed with PBS. The cells were then fixed with 4% paraformaldehyde and blocked with 1% BSA in Dulbecco's PBS for 30 min. Next, the cells were incubated overnight with RP215 at 4 °C. After washing, the cells were incubated with a secondary antibody. The images were captured using a confocal microscope (Leica, Wetzlar, Germany). All cell images were captured under the same experimental conditions. Due to the weak adherence of HEK293 cells, flow cytometry was used to evaluate the surface levels of SIA‐CIgG. Cells were collected without trypsin digestion. After washing with PBS, the cells were incubated with RP215 and a secondary antibody. SIA‐CIgG expression was analyzed using a BD LSRFortessa flow cytometer (BD Biosciences, Franklin Lakes, NJ, USA).

### Vector Constructs and Lentiviral Production

CAR fragments were assembled and inserted into the pHR‐SFFV backbone using the Gibson Assembly (New England Biolabs, Ipswich, MA, USA). The CAR vector consists of a human CD8α signal peptide, scFv, human CD8 hinge, human CD28 transmembrane, human 4‐1BB or CD28, human CD3ζ, and was linked to GFP via a P2A ribosomal skip element. A Myc tag was used to assess the efficiency of scFv expression on the T cell membrane. Utilizing the identical method, the fragment that expresses luciferase fused with a T2A‐connected mCherry was assembled into the pHR‐EF1α backbone. The virus‐package plasmids PCMVdR8.91, PMD2.G, and plasmids containing CAR or luciferase were extracted using the QIAprep Spin Miniprep Kit (Qiagen, Hilden, Germany) following the manufacturer's protocol. The lentivirus was generated by delivering the aforementioned plasmids into the HEK293 packaging cell line using calcium phosphate, as previously described.^[^ ^48^ ^]^ The supernatant was harvested at 48 and 72 h post‐transfection, followed by centrifugation at 3000 × g for 5 min to eliminate cellular debris. The lentiviral particles were concentrated following the manufacturer's protocol (Takara, Shiga, Japan).

### Generation and Expansion of CAR‐T Cells

Human peripheral blood mononuclear cells (PBMC) were purchased from Shanghai Aoneng Biotechnology Co., Ltd. (Shanghai, China), and isolated by density gradient centrifugation (STEMCELL Technologies, Vancouver, Canada). T cells were activated in 24‐well non–tissue culture–treated plates precoated with 1 µg mL^−1^ anti‐CD3 and anti‐CD28 monoclonal antibodies (Biolegend, San Diego, CA, USA) for 48 h. T cells were collected and immediately infected with the lentivirus. After ≈12 h, the virus‐containing culture medium was removed. T cells were resuspended and cultured at a concentration of 10^6^ cells mL^−1^ in complete RPMI 1640 supplemented with 100 U mL^−1^ IL‐2 (PeproTech, Cranbury, NJ, USA) for ≈2 weeks for further experiments.

### Flow Cytometry

Before staining with surface antibodies, cells were washed with PBS. Staining was performed in PBS with 2% FBS at 4 °C for 30 min. The cells were then washed and 7AAD dye (BioLegend) was added to distinguish the dead cells. Isotype‐matched controls were used in each experiment. All samples were analyzed using a BD LSRFortessa flow cytometer (BD Biosciences), and the data were processed using FlowJoV10 (https://www.flowjo.com/solutions/flowjo/downloads). Cells were sorted using a BD Aria Fusion cell sorter (BD Biosciences).

### Cytotoxicity Assays

Cytotoxicity assays were performed by using a previously described bioluminescence method.^[^ ^49^ ^]^ 10^4^ target cells were seeded into white‐walled 96‐well flat‐bottom plates (Beyotime Biotechnology, Shanghai, China). Non‐transduced T cells or CAR‐T cells were placed at multiple ratios in 200 µL of cell culture medium devoid of IL‐2. After a 24 h incubation, the culture medium was aspirated and replaced with 50 µL of fresh medium and 50 µL of luciferase assay reagent (Promega, Madison, WI, USA) in each well. The plates were read using a BioTek Citation 5 after 2 min. Cytotoxicity was assessed using the following formula: lysis efficiency (%) = 100 × [(total luminescence of target cells – luminescence of remaining cells)/(total luminescence of target cells)]. Except for erdafitinib and nivolumab (Selleck), other drugs (vorinostat, birinapant, everolimus, cisplatin, and gemcitabine) (MedChemExpress) were dissolved in dimethyl sulfoxide (DMSO) or dimethylformamide (DMF) as instructed to achieve the recommended storage concentrations. The 5637 cells were seeded in a 96‐well plate for 1 day in advance. After 24 h of treatment, the toxicity of the drug was assessed as described above.

### Measurement of Cytokine Production

Non‐transduced T cells or CAR‐T cells were co‐cultured with pre‐seeded target cells at a 2:1 E:T ratio. After 24 h, supernatants were collected. Cytokine levels were measured using an 8‐plex LEGENDplex multi‐analyte Flow Assay Kit (BioLegend). The experimental procedures were performed following the manufacturer's instructions. Analyses were performed using an LSRFortessa flow cytometer and data analysis software (BioLegend). Owing to the relatively low secretion levels of IL‐4, IL‐6, and IL‐10, the corresponding data are not presented.

### Repeated Tumor Antigen Challenge

Non‐transduced T cells and CAR‐T cells were added to pre‐seeded 5637 cells at an E:T ratio of 1:1 in the presence of IL‐2 (100U mL^−1^). After incubation for 48 h, the cells were collected and introduced into newly pre‐seeded 5637 cells. Similarly, the experiments were terminated based on the experimental requirements. For the cell counting experiment, an equivalent volume was collected from each group after each 48‐h cytotoxic assay. Following 7‐AAD staining, CAR‐T cell counts were determined by flow cytometry. CAR‐T cells were analyzed for PD‐1, TIM‐3, and LAG‐3 expression by flow cytometry using the same procedure.

### RNA‐seq and ATAC‐seq

Following the description of the repeated tumor challenge method, CAR‐T cells were sorted co‐cultured with 5637 cells for seven rounds (14 days). Unstimulated CAR‐T cells were simultaneously cultured and sorted. RNA isolation, library building, quality control, and sequencing were performed by Novogene Co., Ltd. (Beijing, China). ATAC‐seq library preparation was performed according to the protocol provided by Vazyme Biotech Co., Ltd. (Nanjing, China). Subsequently, the library was sequenced by Novogene Co. Ltd. Three biological replicates from three healthy individuals were used for RNA‐seq and ATAC‐seq analyses.

For the raw RNA‐seq data, clean reads were obtained after data filtering, sequencing error rate checks, and GC content distribution checks. HISAT2 software (http://daehwankimlab.github.io/hisat2/) was used to align clean reads to the reference genome, hg38, obtaining the positioning information of reads on the reference genome.^[^ ^50^ ^]^ Subsequently, samtools converted the sam format files to bam format files.^[^ ^51^ ^]^ Finally, featureCounts tool from the subread software was used for quantitative analysis.^[^ ^52^ ^]^ For the raw data of ATAC‐seq, clean reads were aligned to the hg38 reference genome using bowtie2.^[^ ^53^ ^]^ Picard was employed to remove duplicate reads, and samtools was used to remove mitochondrial DNA. Finally, the macs2 software was used to call peaks with a q‐value threshold of 0.01, and ChIPSeeker was used to annotate the peaks.^[^ ^54^ ^]^

The count matrix was converted to a TPM matrix and used the “DESeq2” package in R to perform differential gene expression analysis between the two groups.^[^ ^55^ ^]^ Genes with p‐value ≤ 0.05 and |log2FC| ≥ 2 were defined as differentially expressed. MCP counters based on the transcriptomic data were used to quantitatively assess the abundance of cell subpopulations. The T cell functional gene set scores were calculated using MCP‐counters.^[^ ^56^ ^]^ Heatmaps of chromatin accessibility in the gene body within 2 kb of the transcription start site (TSS) were generated using the R heatmap package. Peak signals of the same gene from the same sample overlapped, and differential analysis of peak signals between the two groups was performed using the DESeq2 package. Peaks with *p*‐value ≤ 0.05 and |log2FC| ≥ 1.5 were considered differentially accessible genes. Volcano plots were constructed using the ggplot2 package in R. Metascape was used for gene enrichment analysis.^[^ ^57^ ^]^

### Mouse Tumor Xenograft Models

Immunocompromised NSG male mice were purchased from Beijing Vitalstar Biotechnology Co., Ltd. (Beijing, China). For xenograft experiments, 2 million 5637 cells expressing luciferase in 100 µL PBS were inoculated subcutaneously in 4–5‐week‐old NSG mice. After 7 days of tumor growth, the mice were divided into four groups based on tumor size and luminescence values. Two groups of mice were intratumorally injected with 1 × 10^7^ non‐transduced T cells or SIA‐CIgG CAR‐T cells (the positivity rate for CAR‐T cells was 50%). Mice in the two combined treatment groups were injected intraperitoneally with 2.0 mg kg^−1^ vorinostat (PBS vehicle) daily for five consecutive days, besides T cells. The tumor size of the mice was monitored twice weekly. Tumor bioluminescence imaging was performed before T cell injection and 10 days after injection.

### Statistical Analysis

All experiments were performed independently at least thrice. Data are shown as mean ± standard deviation (SD). Statistical analyses were performed using GraphPad Prism version 8 (Graphpad Software, San Diego, CA, USA) and SPSS version 27 (IBM Corp., Armonk, NY, USA). Comparisons between two groups were performed using a two‐tailed student's *t*‐test. For multiple‐group comparisons, one‐way analysis of variance (ANOVA) was used to determine whether there were differences among the groups. The P value of each experiment is included in the figure legends, *p* < 0.05 indicated statistically significant differences and “ns” means “not significant” (*p* > 0.05).

## Conflict of Interest

The authors declare no conflict of interest.

## Author Contributions

M.D., J.L., and C.Q. contributed equally to this work. T.X., P.W., X.Q., and M.D. designed the study. M.D. performed the experiments, analyzed and interpreted the data, and wrote the manuscript. J.L. analyzed the RNA‐seq and ATAC‐seq data. C.Q. coordinated the project and provided assistance in the immunohistochemistry and animal experiments. F.Y. provided experimental technical support and advice. Y.D. provided conceptual guidance. T.X., P.W., and X.Q. offered guidance in research and manuscript editing.

## Supporting information

Supporting Information

## Data Availability

The data that support the findings of this study are available from the corresponding author upon reasonable request.
