# Supplementary material for: Novel CAR‐T Cells Specifically Targeting SIA‐CIgG Demonstrate Effective Antitumor Efficacy in Bladder Cancer
Source: Adv Sci (Weinh). 2024 Aug 23;11(40):2400156. doi: 10.1002/advs.202400156 (PMC11516049; doi:10.1002/advs.202400156)
Supplement: Supplementary file 1 — Supporting Information [file ADVS-11-2400156-s001.docx]

Table S1. The expression of SIA-CIgG in tissues from bladder cancer (BC) and healthy bladder specimens

|  | Total | SIA-CIgG expression | | | |
| --- | --- | --- | --- | --- | --- |
|  |  | Negative | Low | Medium | High |
| BC | 63 | 16 (25.4%) | 20 (31.7%) | 11 (17.5%) | 16 (25.4%) |
| Bladder | 17 | 8 (47.1%) | 9 (52.9%) | 0 | 0 |

Table S2. The expression of SIA-CIgG in tissues from BC

|  | Total | SIA-CIgG expression | | | |
| --- | --- | --- | --- | --- | --- |
|  |  | Negative | Low | Medium | High |
| BC | 140 | 35 (25.0%) | 50 (35.7%) | 22 (15.7%) | 33 (23.6%) |

Table S3. The doses of the low, medium, and high concentrations of drugs used in this study.

|  | Concentration | | |
| --- | --- | --- | --- |
|  | Low | Mediate | High |
| Vorinostat | 0.1 | 1 | 5 |
| Birinapant | 0.01 | 0.1 | 1 |
| Nivolumab | 5 | 10 | 20 |
| Everolimus | 0.001 | 0.01 | 0.1 |
| Cisplatin | 0.2 | 1 | 5 |
| Gemcitabine | 0.005 | 0.1 | 1 |
| Erdatifinb | 0.5 | 1 | 5 |

Except for Nivolumab (μg/ml), the units for all other drugs are μM.

**
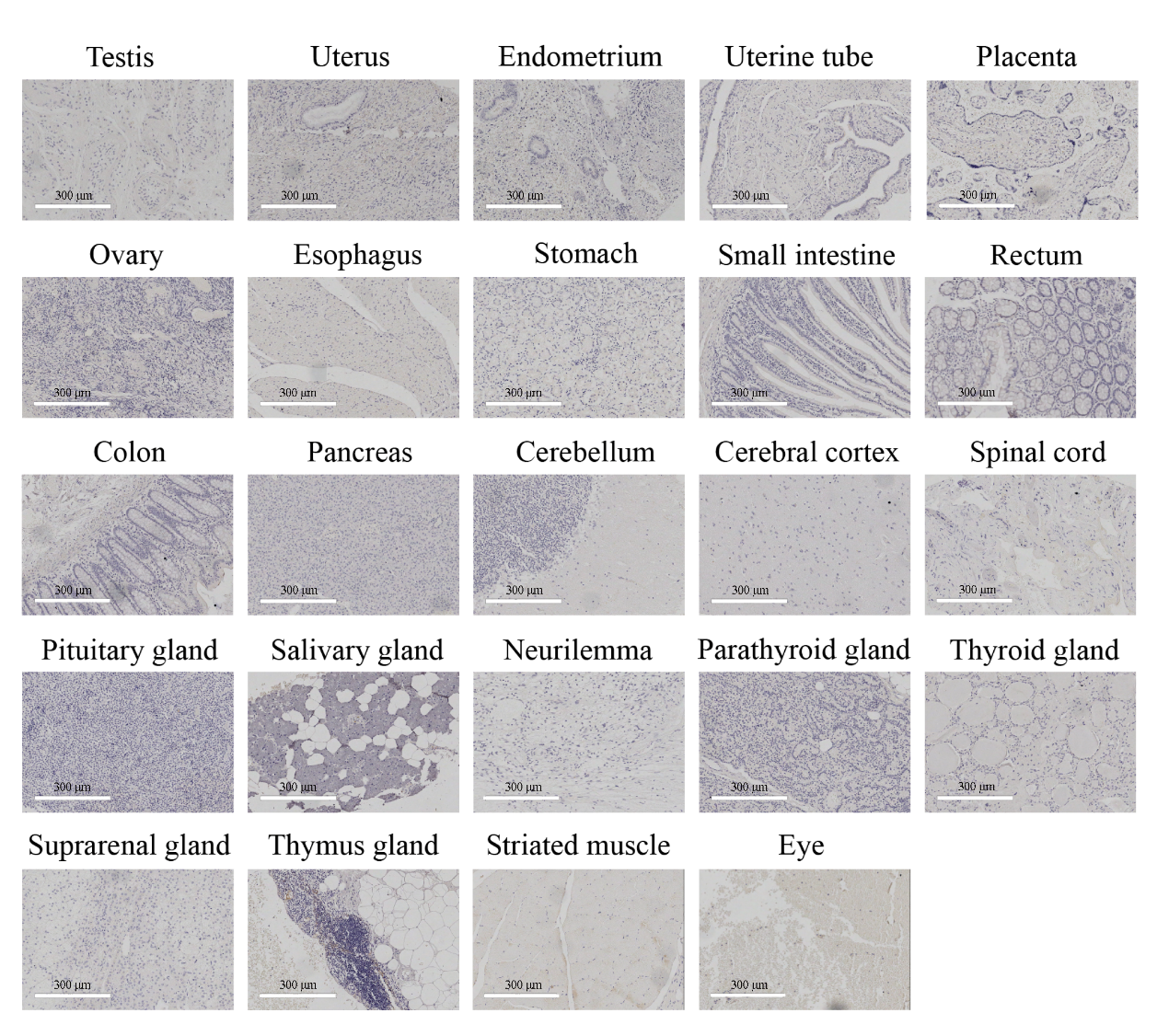
**

**Supplementary Figure S1.** Immunohistochemistry images showcasing the expression profile of SIA-CIgG across 24 distinct normal tissues. Magnification, 10×. Scale bar, 300 μm.

**
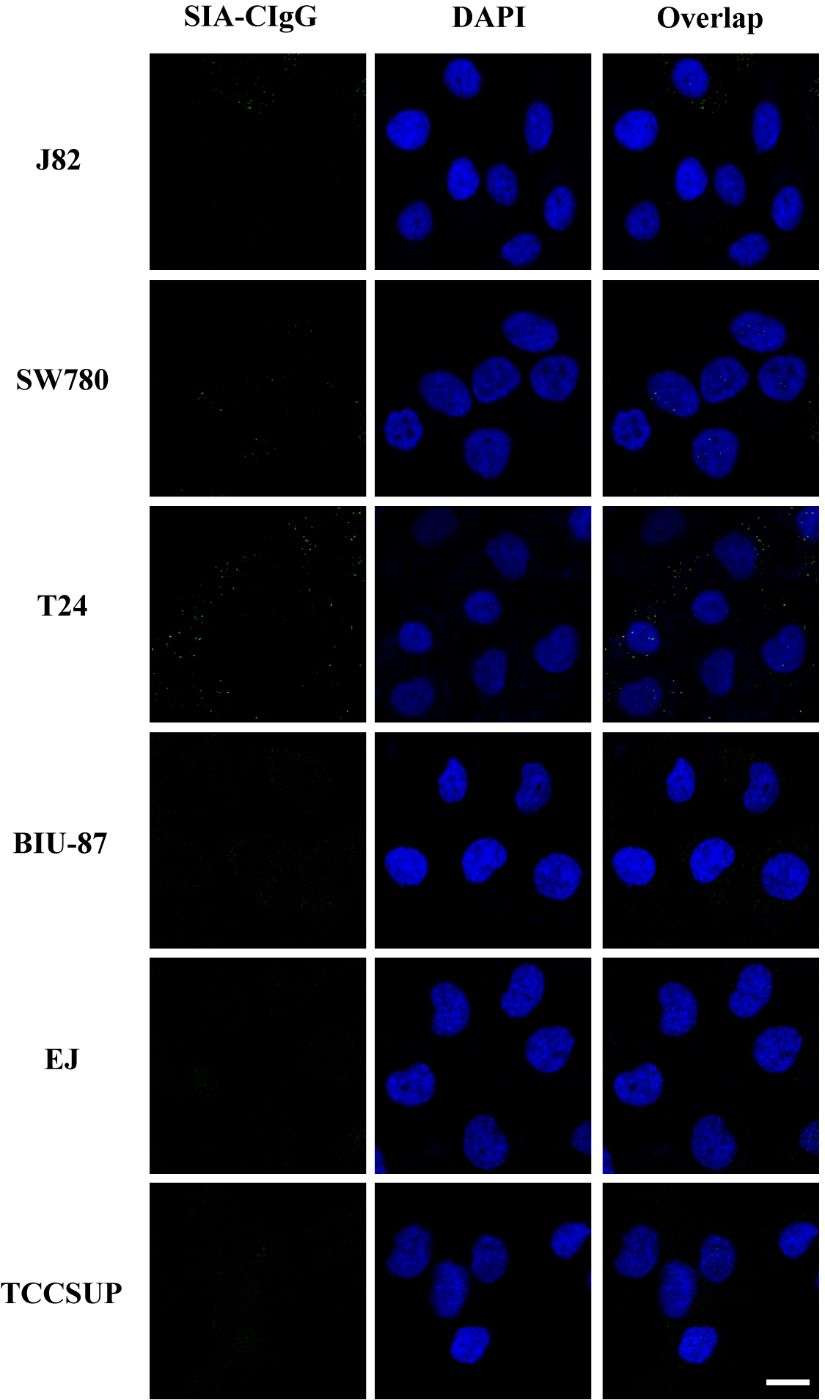
**

**Supplementary Figure S2.** Evaluation of surface expression levels of SIA-CIgG in six BC cell lines using cell immunofluorescence staining. Scale bar, 10 μm.

**
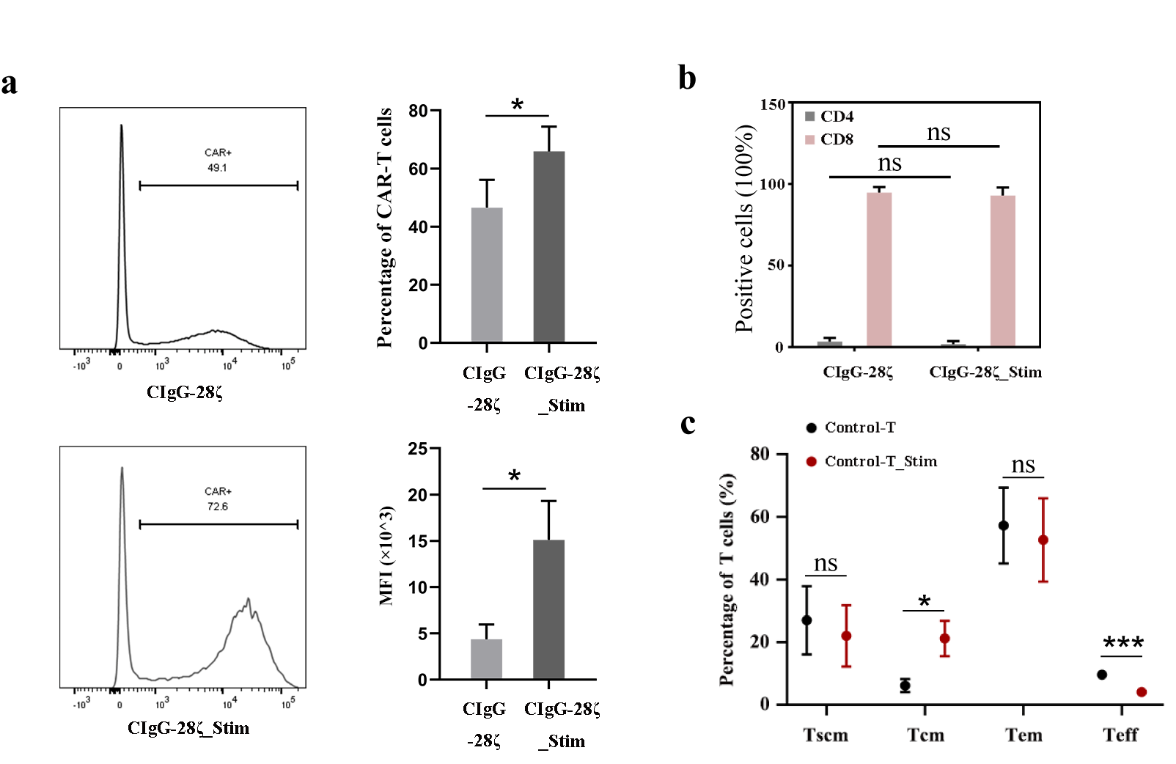
**

**Supplementary Figure S3.** After viral infection, T cells underwent three rounds of tumor challenges without prior sorting. **a,** The proportion of SIA-CIgG CAR-T cells in both the stimulated and non-stimulated cell populations was measured, along with their respective MFI. **b,** The percentages of CD8+ and CD4+ T cells were measured. The SIA-CIgG CAR-T cell population was predominantly CD8+ T cells, with few CD4+ T cells. **c,** The alterations in the proportions of Tscm, Tcm, Tem, and Teff subsets in the nontransfected CAR-T cell population were examined. *P < 0.05, **P < 0.01, ***P < 0.001, “ns” means “not significant” (P > 0.05).

**
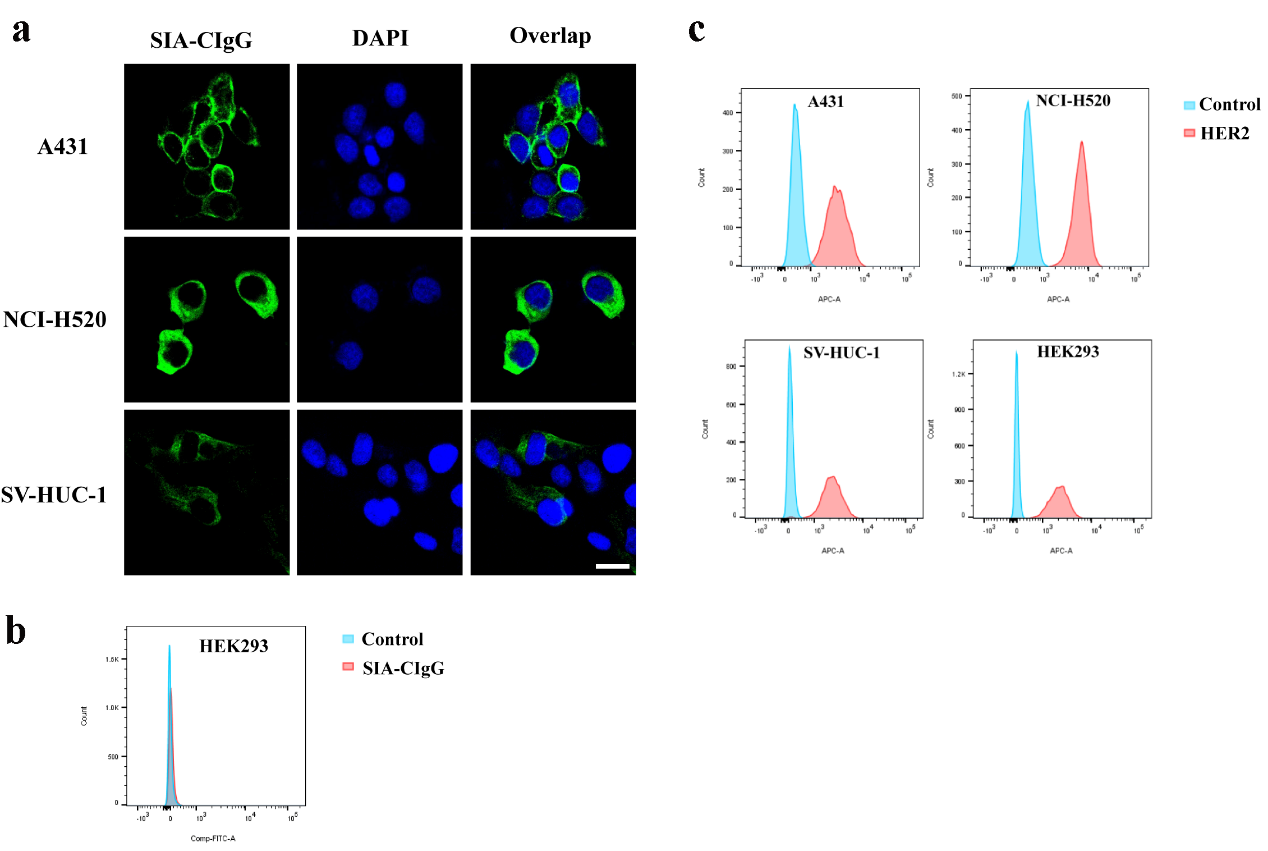
**

**Supplementary Figure S4. a,** Detection of surface expression levels of SIA-CIgG in A431, NCI-H520, and SV-HUC-1 cells using cell immunofluorescence staining. Scale bar, 10 μm. **b,** Assessment of SIA-CIgG surface expression levels in HEK293 cells through flow cytometry. **c,** The expression levels of HER2 on the surface of various cell lines were assessed using flow cytometry.
